# Clinicopathological Characteristics and Prognosis in Endometrial Cancer With Bone Metastasis: A SEER-Based Study of 584 Women

**DOI:** 10.3389/fonc.2021.694718

**Published:** 2021-07-01

**Authors:** Hejia Hu, Zhan Wang, Miaofeng Zhang, Feng Niu, Qunfei Yu, Ying Ren, Zhaoming Ye

**Affiliations:** ^1^ Department of Orthopedic Surgery, The Second Affiliated Hospital, Zhejiang University School of Medicine, Hangzhou, China; ^2^ Orthopedics Research Institute of Zhejiang University, Hangzhou, China; ^3^ Key Laboratory of Motor System Disease Research and Precision Therapy of Zhejiang Province, Hangzhou, China; ^4^ Department of Orthopedics, Ningbo Municipal Hospital of TCM, Ningbo, China

**Keywords:** endometrial cancer, bone metastasis, clinicopathological characteristics, survival, risk factors

## Abstract

**Purpose:**

Bone metastasis from endometrial cancer (EC) is rare and poorly described. The purpose of the present study was to investigate the correlation between the clinically accessible factors and survival time among EC patients with bone metastasis.

**Patients and Methods:**

We retrospectively identified and reviewed EC patients with bone metastasis from 2010 to 2016, based on the Surveillance, Epidemiology and End Results (SEER) database. Univariable and multivariable Cox regressions were applied to evaluate the effects of clinical variables on survival. Kaplan–Meier plots were used to visually demonstrate the correlation between independent risk factors and survival.

**Results:**

Clinical data of 584 EC patients with bone metastasis from the SEER database were analyzed. EC patients with bone metastasis experienced extremely poor survival, with 1-year overall survival (OS) and cancer-specific survival (CSS) rates 33.8 and 35.8%, respectively. Variables associated with OS and CSS in the univariable analysis included race, tumor grade, tumor subtype, tumor size, lung, liver and brain metastases, surgery, radiotherapy, and chemotherapy. In the multivariable analysis, tumor grade, tumor subtype, liver and brain metastases, local surgery, and systemic chemotherapy remained independent risk factors for OS and CSS. However, local radiotherapy was an independent predictor of OS, not CSS.

**Conclusions:**

We identified several factors affect the survival of EC patients with bone metastasis, which is useful for clinicians to assess patients’ outcomes. Our study supports surgery and radiotherapy of primary EC, and systemic chemotherapy for prolonging survival among EC patients with bone metastasis, which lays a solid foundation for defining optimal treatment strategy in this specific cohort.

## Introduction

As the most common malignancy of female endometrium, endometrial cancer (EC) incidence rate has been rapidly rising in recent years (1, 2). With the rapid development of imaging techniques and treatment of EC, the incidence of bone metastasis is also increasing. Compared with other tumors, bone metastasis is relatively uncommon in EC, with an incidence 0.6% (3). Among patients in stage IV EC, bone metastasis occurred in 6.8% of patients (4). Distant organ metastasis usually predicts a poor prognosis (5). Although 5-year overall survival (OS) of EC was estimated around 80% in patients without metastatic disease (1, 2), metastatic EC patients experienced extremely poor prognosis with 5-year OS less than 20% (3, 4). Common treatment methods for EC include surgery, radiotherapy, as well as systemic therapies. However, the standard management of EC with bone metastasis is unknown.

Some studies reported risk factors associated with survival among EC, including tumor stage, histological type, adjuvant chemotherapy, etc. (6, 7). However, EC patients with bone metastasis constitute a heterogeneous cohort, survival predictors and appropriate treatments of them remain unknown. Additionally, the Surveillance, Epidemiology and End Results (SEER) database used in the study, is widely used in rare tumor entities. This study was aimed to reveal the clinicopathologic features and assess the risk factors associated with prognosis of this rare population.

## Material And Methods

### Study Population

In this retrospective study, we included patients diagnosed as EC with bone metastasis between 2010 and 2016. Clinical data regarding EC with bone metastasis were collected from the SEER database, including basic clinicopathological information and treatment methods. As a large population-based database, the SEER represents nearly 30% of the US population and provides a free tool for clinical study of malignant tumors, which covers 20 geographic areas in the USA (8).

We used two keywords Primary site C54.1-Endometrium and SEER Combined Mets at DX-bone (2010+) to retrieve EC cases with bone metastasis in the SEER database. In addition, we only included cases with pathological diagnosis. According to the International Classification of Diseases for Oncology, Third Edition (ICD-O-3), we categorized EC with bone metastasis into three histological subtypes: endometrioid subtype (8380), non-endometrioid subtype (8000, 8010, 8013, 8020, 8041, 8045– 8046, 8050, 8070, 8140, 8246, 8255, 8260, 8310, 8323, 8441, 8460–8461, 8480, 8560, 8570, 8574), and sarcoma subtype (8800, 8805, 8890,8900, 8930–8931,8950,8980). Surgery or radiotherapy in the current research refers to the primary EC (9).

### Statistical Analyses

We completed all statistical analyses by IBM SPSS Statistics 22. We defined cancer-specific survival (CSS) as the interval from diagnosis till death due to EC (10, 11). We used the univariable Cox regression model to screen for statistically significant indicators associated with OS and CSS. Then, we performed multivariable Cox regression model to confirm independent predictors of OS and CSS. Kaplan–Meier plots were used to visually demonstrate the correlation between independent risk factors and survival. Hazard ratios (HRs) and 95% confidence interval (CI) were used to show the impact of variables on survival during Cox regression analyses. Analysis variables with bilateral *p* less than 0.05 were considered statistically significant.

## Results

### Clinicopathological Characteristics

Of the 584 EC patients with bone metastasis identified, more than two-thirds (71.4%) of patients were white. Average and median age are both 64 years old. Two hundred sixteen (37.0%) patients were aged less than 60 years old and 368 (63.0%) patients were aged over 60 years old. Tumor grade was defined as low grade (n = 76, 13.0%), high grade (n = 329, 56.3%), and unknown grade (n = 179, 30.7%). Histological subtype distribution was endometrioid 36.1%, non-endometrioid 51.5%, and sarcoma 12.3%. Sixty-five (11.1%), 137 (23.5%), and 68 (11.6%) of the patients had tumor size <5 cm, 5–10 cm, and >10 cm, respectively. Lung metastasis accounted for 46.2%, liver metastasis accounted for 23.8%, and brain metastasis accounted for 7.2%. About two-fifths (40.8%) of the patients were married. Surgery was performed for 213 (36.5%) patients, radiotherapy was performed for 252 (43.2%), and chemotherapy was performed for 314 (53.8%). One-year OS and CSS rate for all patients was 33.8 and 35.8%, respectively (**Table 1**).

**Table 1:** Baseline characteristics of 584 endometrial cancer bone metastasis.

Variable	Value
**Race**	
White	417 (71.4%)
Black	100 (17.1%)
Others	67 (11.5%)
**Age (years)**	
≤60	216 (37.0%)
>60	368 (63.0%)
Mean	64
Median	64
**Tumor grade**^**a**^	
Low grade	76 (13.0%)
High grade	329 (56.3%)
Unknown	179 (30.7%)
**Tumor subtype**	
Endometrioid	211 (36.1%)
Non-endometrioid	301 (51.5%)
Sarcoma	72 (12.3%)
**Tumor size (cm)**	
<5	65 (11.1%)
5–10	137 (23.5%)
>10	68 (11.6%)
Unknown	314 (53.8%)
**Surgery**	
Yes	213 (36.5%)
No	371 (63.5%)
**Radiotherapy**	
Yes	252 (43.2%)
No	332 (56.8%)
**Chemotherapy**	
Yes	314 (53.8%)
No	270 (46.2%)
**Brain metastasis**	
No	542 (92.8%)
Yes	42 (7.2%)
**Liver metastasis**	
No	445 (76.2%)
Yes	139 (23.8%)
**Lung metastasis**	
No	314 (53.8%)
Yes	270 (46.2%)
**Marital status**	
Married	238 (40.8%)
Others	308 (52.7%)
Unknown	38 (6.5%)
**Dead**	
Yes	458 (78.4%)
No	126 (21.6%)
**1-year OS rate**	33.80%
**1-year CSS rate**	35.80%

a Low grade, ICD-O-3 Grade 1 (well differentiated) and Grade 2 (moderately differentiated); High grade, ICD-O-3 Grade 3 (poorly differentiated) and Grade 4 (undifferentiated anaplastic); OS, overall survival; CSS, cancer-specific survival.

### Univariable Cox Regression Analysis

**Figure 1:**
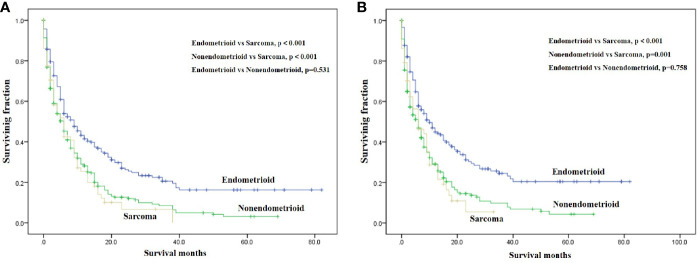
Kaplan-Meier method estimated OS **(A)** and CSS **(B)** in endometrial cancer bone metastasis stratified by tumor subtype. (OS, overall survival; CSS, cancer-specific survival).

**Figure 2:**
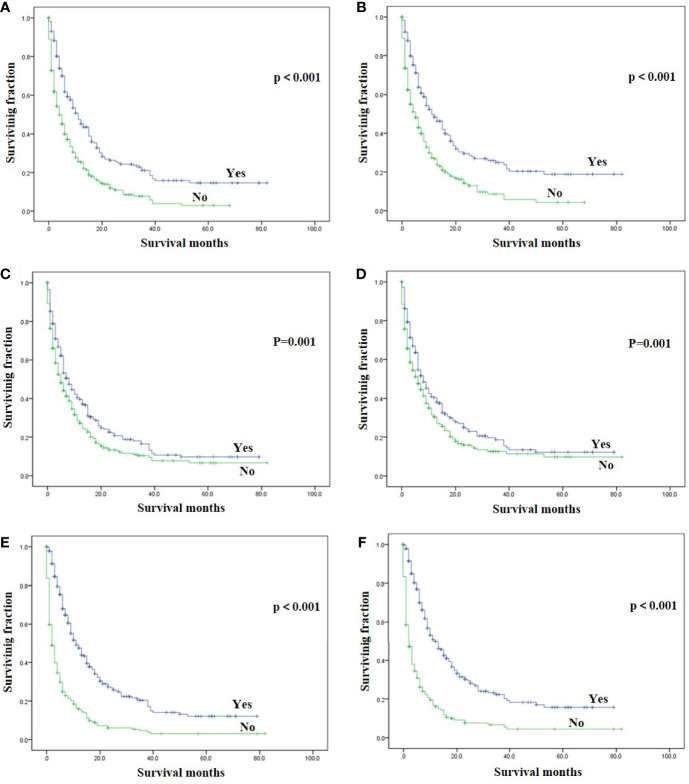
Kaplan-Meier method estimated OS and CSS in endometrial cancer bone metastasis stratified by treatment methods. **(A)** OS stratified by surgery; **(B)** CSS stratified by surgery; **(C)** OS stratified by radiotherapy; **(D)** CSS stratified by radiotherapy; **(E)** OS stratified by chemotherapy; **(F)** CSS stratified by chemotherapy. (OS, overall survival; CSS, cancer-specific survival).

On univariable analysis, black race, high tumor grade, non-endometrioid, and sarcoma subtype, tumor size >10 cm, the presence of lung, liver and brain metastases, no surgery, no radiotherapy, and no chemotherapy were significant predictors for decreased OS and CSS (**Table 2**). There was no difference in OS and CSS by age and marital status (**Table 2**). The Kaplan–Meier curve plots displayed that patients with endometrioid subtype had the best survival, followed by non-endometrioid and sarcoma subtypes (**Figure 1**, p < 0.05). Moreover, surgery, radiotherapy, and chemotherapy had a significant survival benefit for patients (**Figure 2**, p < 0.05).

**Table 2:** Univariate Cox analysis of variables in endometrial cancer bone metastasis.

Variable	OS		CSS	
	HR (95% CI)	*P*	HR (95% CI)	*P*
**Race**				
White	1		1	
Black	1.497 (1.180–1.899)	0.001	1.448 (1.104–1.899)	0.007
Others	0.800 (0.589–1.086)	0.153	0.790 (0.564–1.106)	0.17
**Age (years)**				
≤60	1		1	
>60	1.079 (0.891–1.307)	0.437	1.022 (0.829–1.262)	0.836
**Tumor grade**^**a**^				
Low grade	1		1	
High grade	1.571 (1.165–2.117)	0.003	1.717 (1.246–2.365)	0.001
**Tumor subtype**				
Endometrioid	1		1	
Non-endometrioid	1.566 (1.277–1.920)	<0.001	1.683 (1.340–2.115)	<0.001
Sarcoma	1.696 (1.252–2.297)	<0.001	1.757 (1.243–2.483)	<0.001
**Tumor size (cm)**				
<5	1		1	
5–10	1.159 (0.811–1.657)	0.418	1.152 (.767–1.731)	0.495
>10	1.773 (1.188–2.647)	0.005	1.848 (1.175–2.905)	0.008
**Surgery**				
Yes	1		1	
No	1.836 (1.508–2.235)	<0.001	1.856 (1.487–2.316)	<0.001
**Radiotherapy**				
Yes	1		1	
No	1.345 (1.116–1.620)	0.002	1.305 (1.060–1.605)	0.012
**Chemotherapy**				
Yes	1		1	
No	2.669 (2.213–3.219)	<0.001	2.761 (2.239–3.407)	<0.001
**Brain metastasis**				
No	1		1	
Yes	1.689 (1.205–2.367)	0.002	1.633 (1.135–2.351)	0.008
**Liver metastasis**				
No	1		1	
Yes	1.686 (1.373–2.072)	<0.001	1.835 (1.462–2.305)	<0.001
**Lung metastasis**				
No	1		1	
Yes	1.217 (1.013–1.462)	0.036	1.250 (1.018–1.535)	0.033
**Marital status**				
Married	1		1	
Others	1.099 (0.907–1.331)	0.337	1.065 (0.860–1.320)	0.564
Unknown	1.044 (0.713–1.528)	0.825	1.045 (0.679–1.610)	0.84

a Low grade, ICD-O-3 Grade 1 (well differentiated) and Grade 2 (moderately differentiated); High grade, ICD-O-3 Grade 3 (poorly differentiated) and Grade 4 (undifferentiated anaplastic); OS, overall survival; CSS, cancer-specific survival.

### Multivariable Cox Regression Analysis

Using multivariable analysis, high tumor grade, non-endometrioid and sarcoma subtype, the presence of liver and brain metastases, no surgery, and no chemotherapy were significant predictors of worsened OS and CSS (**Table 3**). Radiotherapy is an independent predictor for OS, not CSS (**Table 3**). No difference was observed in OS and CSS by race, tumor size, and lung metastasis (**Table 3**).

**Table 3:** Multivariate Cox analysis of variables in endometrial cancer bone metastasis.

Variable	OS		CSS	
	HR (95% CI)	*P*	HR (95% CI)	*P*
**Race**				
White	1		1	
Black	1.083 (0.848–1.384)	0.524	1.065 (0.806–1.407)	0.659
Others	0.838 (0.611–1.151)	0.275	0.803 (0.568–1.137)	0.217
**Tumor grade**^**a**^				
Low grade	1		1	
High grade	1.772 (1.284–2.446)	0.001	1.662 (1.159–2.383)	0.006
**Tumor subtype**				
Endometrioid	1		1	
Non-endometrioid	1.444 (1.130–1.845)	0.003	1.444 (1.130–1.845)	0.003
Sarcoma	2.031 (1.402–2.944)	<0.001	2.031 (1.402–2.944)	<0.001
**Tumor size (cm)**				
<5	1		1	
5–10	1.238 (0.856–1.789)	0.257	1.363 (0.890–2.088)	0.155
>10	1.450 (0.958–2.193)	0.079	1.509 (0.945–2.411)	0.085
**Surgery**				
Yes	1		1	
No	1.714 (1.378–2.131)	<0.001	1.763 (1.383–2.247)	<0.001
**Radiotherapy**				
Yes	1		1	
No	1.256 (1.032–1.527)	0.023	1.228 (0.985–1.530)	0.068
**Chemotherapy**				
Yes	1		1	
No	3.149 (2.577–3.848)	<0.001	3.323 (2.654–4.162)	<0.001
**Brain metastasis**				
Yes	1		1	
No	1.800 (1.265–2.564)	0.001	1.803 (1.234–2.653)	0.002
**Liver metastasis**				
No	1		1	
Yes	1.617 (1.300–2.011)	<0.001	1.744 (1.371–2.218)	<0.001
**Lung metastasis**				
No	1		1	
Yes	1.057 (0.875–1.278)	0.563	1.059 (0.856–1.311)	0.595

a Low grade, ICD-O-3 Grade 1 (well differentiated) and Grade 2 (moderately differentiated); High grade, ICD-O-3 Grade 3 (poorly differentiated) and Grade 4 (undifferentiated anaplastic); OS, overall survival; CSS, cancer-specific survival.

## Discussion

Although metastatic EC patients experienced poor prognosis, few researches explored risk factors of survival and effective treatments for them. We first performed the largest study for EC patients with bone metastasis to reveal their clinical characteristics, prognosis, and risk factors affecting prognosis. Our results validated that tumor grade, tumor subtype, liver and brain metastases were independent predictor associated with the OS and CSS. In terms of treatment methods, surgery, radiotherapy, and chemotherapy were positive independent predictors of OS, while surgery, and chemotherapy were positive independent predictors of CSS. The most important clinical significance of this study is to guide clinicians to better evaluate the survival of EC patients with bone metastasis and provide appropriate treatments.

The prognosis of EC with bone metastasis was extremely poor, with 1-year OS and CSS rate 33.8 and 35.8%, respectively. Therefore, the prognosis of these patients has a great room for improvement. It is very important and necessary to explore the risk factors affecting the prognosis of these patients. Race was not a significant independent risk factor of survival in this study, unlike many previous studies that it had an impact on the prognosis of EC (12, 13). This may be due to the fact that many previous studies have focused on all patients with EC, but this study only focused on EC patients with bone metastasis. Our univariable analysis also showed that age was not correlated with OS or CSS, which was not congruent with many previous studies including metastatic EC (3, 14, 15). Further researches are required to confirm our finding. This study found that tumor grade was an independent prognostic factor for OS and CSS, which was consistent with others (16–18). Our multivariable analysis showed that patients with non-endometrioid and sarcoma subtypes had worse survival than those with endometrioid subtype, consistent with the findings reported by Liu et al. (4). Although tumor size was significant in a univariable analysis, this variable did not remain significant in multivariable analysis. Many previous studies found tumor size was an important independent predictor of EC patients with lower stage (19, 20). Primary tumor size may not play a significant role in prognosis in metastatic patients. Additionally, Caner Çakır et al. (21)reported that tumor size did not have prognostic value in EC patients with stage I or II. In the current study, no association between survival and marital status was found.

Our analysis found that lung was the most common metastatic organ (46.2%) in EC patients with bone metastasis, compared with other metastatic organs. In contrast to previous studies (7, 22), lung metastasis did not remain significant in multivariable analysis. Consistent with previous studies (3, 22), liver or brain metastasis were independent predictors of decreased OS and CSS in the multivariable analysis. Therefore, aggressive management of liver and brain metastases may be helpful in prolonging outcomes in EC patients with bone metastasis.

Traditional medical treatments for EC patients include surgery, radiotherapy, and chemotherapy (12, 23, 24). However, few data factually support their survival benefits in EC patients with bone metastasis. This study emphasized that good outcomes were associated with surgery and radiotherapy of primary tumors, and systemic chemotherapy, which agrees with the previous findings (12, 22). We first found that surgery or radiotherapy of primary tumors were independent predictors associated with increased OS. Although radiotherapy did not remain a significant risk factor in CSS, it can be used for palliative treatment of such patients (6, 25, 26). Multimodality therapy is strongly recommended for EC patients with bone metastasis.

Several limitations should be noted in this study. First, the retrospective nature of this study cannot be ignored. Second, clinical data on other systemic therapies are not available in the SEER database. Third, the types of surgery or radiotherapy are still not available in SEER database. Despite the above disadvantages of the SEER database, this cancer database provides clinicians a very useful tool for clinical cancer research.

## Conclusions

We identified several factors affect the survival of EC patients with bone metastasis, including tumor grade, tumor subtype, liver and brain metastases, surgery, radiotherapy, and chemotherapy. This study is helpful for tailoring treatment regimen for EC patients with bone metastasis. However, further validation of our findings is needed in prospective, multicenter, randomized controlled studies.

## Data Availability Statement

The raw data supporting the conclusions of this article will be made available by the authors, without undue reservation.

## Ethics Statement

The studies involving human participants were reviewed and approved by the Ethics Committee of Second Affiliated Hospital, Zhejiang University School of Medicine. Written informed consent for participation was not required for this study in accordance with the national legislation and the institutional requirements.

## Author Contributions

ZW, YR, and ZY conceived and designed the study. HH, ZW, and MZ collected the data. HH, ZW, MZ, FN, and QY performed the statistical analysis. HH and ZW wrote the manuscript. ZW, YR, and ZY revised it. All authors contributed to the article and approved the submitted version.

## Funding

This work was supported by the National Natural Science Foundation of China (81872173, 82072959), Natural Science Foundation of Zhejiang Province (LD21H160002), and Medical and Health Science and Technology Plan of Department of Health of Zhejiang Province (WKJ-ZJ-1821).

## Conflict of Interest

The authors declare that the research was conducted in the absence of any commercial or financial relationships that could be construed as a potential conflict of interest.
